# The *Plasmodium falciparum* Hsp70-x chaperone assists the heat stress response of the malaria parasite

**DOI:** 10.1096/fj.201901741R

**Published:** 2019-11-14

**Authors:** Jemma Day, Armin Passecker, Hans-Peter Beck, Ioannis Vakonakis

**Affiliations:** * Department of Biochemistry, University of Oxford, Oxford, United Kingdom;; † Swiss Tropical and Public Health Institute, Basel, Switzerland;; ‡ University of Basel, Basel, Switzerland

**Keywords:** PfHsp70-x, protein structure, chaperone-cochaperone interactions, chaperone inhibition

## Abstract

*Plasmodium falciparum* is the most lethal of human-infective malaria parasites. A hallmark of *P. falciparum* malaria is extensive remodeling of host erythrocytes by the parasite, which facilitates the development of virulence properties such as host cell adhesion to the endothelial lining of the microvasculature. Host remodeling is mediated by a large complement of parasite proteins exported to the erythrocyte; among them is a single heat shock protein (Hsp)70–class protein chaperone, *P. falciparum* Hsp70-x (PfHsp70-x). PfHsp70-x was previously shown to assist the development of virulent cytoadherence characteristics. Here, we show that PfHsp70-x also supports parasite growth under elevated temperature conditions that simulate febrile episodes, especially at the beginning of the parasite life cycle when most of host cell remodeling takes place. Biochemical and biophysical analyses of PfHsp70-x, including crystallographic structures of its catalytic domain and the J-domain of its stimulatory Hsp40 cochaperone, suggest that PfHsp70-x is highly similar to human Hsp70 chaperones endogenous to the erythrocyte. Nevertheless, our results indicate that selective inhibition of PfHsp70-x function using small molecules may be possible and highlight specific sites of its catalytic domain as potentially of high interest. We discuss the likely roles of PfHsp70-x and human chaperones in *P. falciparum* biology and how specific inhibitors may assist us in disentangling their relative contributions.—Day, J., Passecker, A., Beck, H.-P., Vakonakis, I. The *Plasmodium falciparum* Hsp70-x chaperone assists the heat stress response of the malaria parasite.

Despite substantial progress during the last 2 decades, malaria still remains one of the most lethal infectious diseases in tropical countries, causing an estimated 435,000 deaths a year, primarily in sub-Saharan Africa and southeast Asia (1). Recently, the global fight against malaria appears to have stalled, as evidenced by slightly increasing numbers of malaria deaths since 2015 alongside well-documented incidences of resistance to frontline therapeutics. The vast majority of malaria deaths are caused by the apicomplexan parasite *Plasmodium falciparum* (2). Key to *P. falciparum* lethality is the extensive remodeling of its host cell, the human erythrocyte, for which it employs a larger proportion of its proteome (∼10%, ∼500 proteins) than any other human-infective malaria parasite (3). Erythrocyte remodeling is essential for *P. falciparum* survival, notably as it increases uptake of nutrients, ensures ion homeostasis, and alters host cell structure and rigidity (4–7). The later alterations, coupled to the formation of protrusions (knobs) on the erythrocyte membrane, promote host cell clumping (rosetting) and strong cytoadherence to the endothelium *via* the parasite virulence factor *P. falciparum* erythrocyte membrane protein 1 (PfEMP1) (8), which localizes in knobs. Erythrocyte rosetting and cytoadherence prevent passage of infected erythrocytes through the spleen, where they would be destroyed, but also lead to disruption of blood flow in the microvasculature causing oxygen deprivation in tissues, inflammation, and organ damage (9, 10). Thus, understanding the mechanisms that support *P. falciparum* survival and virulence is imperative as we seek to combat malaria.

The *P. falciparum* genome encodes numerous molecular chaperones thought to aid in coping with the stress of infection (11, 12). The latter includes both periodic heat shock of parasites during febrile episodes and oxidative stress by free radical groups, which are generated by iron-containing heme released upon hemoglobin degradation (13). Heat shock protein (Hsp)70 chaperones, widespread in all kingdoms of life (14), are known to be key for protein quality control; for assisting protein translocation, folding, and macromolecular complex assembly; and for preventing the aggregation of damaged proteins (15). In the *Laverania* subgenus of malaria parasites, of which *P. falciparum* is the only human-infective member, a single Hsp70 chaperone, Hsp70-x, is exported to the host cell (16). Common to all *Laverania* parasites is an expanded set of exported proteins for host cell remodeling; thus, it is believed that Hsp70-x ensures the correct folding of these proteins, thereby supporting parasite survival and virulence functions. Notably, whereas the genomic locus of *P. falciparum* Hsp70-x (PfHsp70-x; *pf3d7_0831700*) is located at the subtelomeric region of chromosome 8, which is highly vulnerable to breakage (17), loss of PfHsp70-x has not been reported in any clinical isolates of *P. falciparum*, suggesting that this chaperone plays an important role in parasite biology.

In infected erythrocytes, PfHsp70-x primarily colocalizes with the PfEMP1 virulence factor in highly mobile assemblies termed “J-dots” and in membrane cisternae, the “Maurer’s clefts” (16). In addition to PfEMP1 and PfHsp70-x, J-dots contain multiple Hsp40 (also known as DnaJ) cochaperone proteins (18), which can stimulate Hsp70 activity. At least one of these cochaperones, PFA0660w (*pf3d7_0113700*), was shown to preferentially stimulate PfHsp70-x activity compared with a human Hsp70 (19). Because J-dots and Maurer’s clefts mediate PfEMP1 trafficking from the parasite to the erythrocyte surface membrane, PfHsp70-x localization and activity therein suggest a role for this chaperone in PfEMP1 export. Supporting the role of PfHsp70-x in facilitating *P. falciparum* virulence, transgenic parasites lacking PfHsp70-x trafficked PfEMP1 less efficiently to the host cell membrane and showed ∼60% reduction in adhesion of infected erythrocytes to the placental cytoadherence receptor chondroitin sulfate A under simulated blood flow conditions (20).

Beyond J-dots and Maurer’s clefts, PfHsp70-x was also found diffuse in the erythrocyte cytoplasm (16), where it may provide stress relief. Such a stress-relief role would suggest a direct contribution of PfHsp70-x to parasite survival; however, to date no such contribution has been demonstrated. Parasite growth assays under standard culturing conditions found that PfHsp70-x is dispensable for viability (21), whereas PfHsp70-x knockout parasite lines were only slightly more susceptible to oxidative stress and hypoxanthine restriction compared with wild-type *P. falciparum* (20). Notably, the later study did not identify a contribution of PfHsp70-x to parasite survival under heat shock. However, PfHsp70-x knockout parasites had different protein abundance levels compared with wild-type *P. falciparum* for over 70 components, including 1 intraparasitic Hsp70 and 1 Hsp90 chaperone (20). These changes in protein abundance were attributed to parasite selection during the relatively long timeframe of creating PfHsp70-x knockouts, leading to transcriptional adjustments that may ameliorate the effects of losing this gene. Thus, it is likely that the true contribution of PfHsp70-x to parasite survival under stress might have been masked in this earlier study.

Here, we assess the effect of PfHsp70-x loss on parasite viability using a conditional, rapid protein depletion system, which does not allow time for the selection of parasites with compensatory changes. We find that PfHsp70-x assists parasite growth under heat shock conditions comparable to febrile episodes. Interestingly, the PfHsp70-x effect on parasite viability is strongest at the beginning of the intraerythrocytic cycle, which corresponds both to the period when most exported proteins are translocated to the host cell and when patients with malaria would normally exhibit fever. Furthermore, we present here the crystallographic structures of the PfHsp70-x catalytic domain and the chaperone-stimulatory domain of PFA0660w. Together with biochemical and interaction analyses, these structures allow us to assess for the first time the relationship of PfHsp70-x and human chaperones endogenous to the erythrocyte.

## MATERIALS AND METHODS

### PfHsp70-x knockdown, parasite lysates, and Western blot

A *P. falciparum* parasite line in which PfHsp70-x is conditionally down-regulated (PfHsp70-x_glmS_) was kindly gifted by Dr. Vasant Muralidharan (University of Georgia, Athens, GA, USA) (21). Briefly, PfHsp70-x_glmS_ incorporates a GlcN-6-phosphate riboswitch (*glmS*) ribozyme sequence inserted *via* clustered regularly interspaced short palindromic repeats (CRISPR)/CRISPR associated protein 9 (Cas9) in the 3′ end of the PfHsp70-x gene locus, leading to rapid PfHsp70-x mRNA degradation upon addition of glucosamine (GlcN). PfHsp70-x_glmS_ parasites were split and grown with or without 2.5 mM GlcN for 2 cycles. From each condition, 500 μl of trophozoite-stage infected erythrocytes were lysed in 4 ml 0.03% w/v saponin in PBS (137 mM NaCl, 27 mM KCl, 10 mM sodium phosphate pH 7.4) for 10 min on ice, washed in PBS, and subsequently resuspended in 200 μl sample loading buffer (0.255 M Tris-HCl pH 6.8, 50% v/v glycerol, 5% w/v SDS, 2% v/v 2-ME) supplemented with 1× Complete Proteinase inhibitor cocktail (Roche, Basel, Switzerland) and DNAse I (75 μg/ml; MilliporeSigma, Burlington, MA, USA). Samples were sonicated for 5 min at 30 s intervals at high intensity (Bioruptor; Diagenode, Liège, Belgium) and stored at −20°C until further use.

Lysates were boiled at 95°C for 5 min and 10 μl of each sample was loaded onto a 4–12% Novex NuPage Bis-Tris gel and run in 3-(*N*-morpholino)propanesulfonic acid (MOPS) buffer (both from Thermo Fisher Scientific, Waltham, MA, USA) according to the manufacturer’s recommendations. Proteins were blotted using the iBlot 2 (Thermo Fisher Scientific) at program P0, and the membrane was subsequently blocked in 5% v/v milk in 100 mM Tris-HCl, 150 mM NaCl, 43 mM HCl, and 0.1% v/v Tween 20 before being probed with antibodies. Membranes were probed with rabbit anti–Hsp70-x [1:5000 dilution (17)], rat anti–influenza agglutinin (HA; 1:1000 dilution; Roche), and mouse anti–glyceraldehyde 3-phosphate dehydrogenase (1:10,000 dilution; gift from C. Daubenberger, Swiss Tropical and Public Health Institute) as primary antibodies, and goat anti-mouse horseradish peroxidase (HRP; 1:10,000 dilution; Pierce, Rockford, IL, USA), goat anti-rabbit HRP (1:10,000 dilution; Jackson ImmunoResearch Laboratories, West Grove, PA, USA), and goat anti-rat HRP (1:10,000 dilution; Southern Biotech, Birmingham, AL, USA) were used as secondary antibodies.

### Parasite culturing and heat shock

PfHsp70-x_glmS_ parasites (21) were cultured according to standard methods (22) at 5% hematocrit. Parasites were synchronized using sorbitol to initially obtain a pure ring-stage culture. Subsequently, late stage parasites of the same cycle were enriched using a 70% v/v Percoll gradient and incubated on a shaker at 45 rpm to obtain single infections upon reinvasion. Eight hours later, parasites were synchronized again using sorbitol to lyse remaining schizonts and obtain an 8-h time window of early ring-stage parasites. Cultures were split, and 2.5 mM GlcN was added to one culture to induce PfHsp70-x knockdown (23). For the heat shock experiments done 4–12 h postinfection (hpi), sorbitol treatment was performed 4 h after Percoll enrichment to tighten the time window. Following synchronization, parasites were seeded into 6-well plates, starting parasitemia was determined using flow cytometry, and 8 h–long (for the 4–12 hpi) or 12 h–long (all other timepoints) 40°C heat shock was performed at the given timepoints postinvasion, whereas the control was incubated at 37°C for the whole course of the experiment. About 48 h after the initial Percoll enrichment, the parasitemia was measured using flow cytometry when Giemsa smears showed that all schizonts had ruptured and parasites were in the ring stage of the consecutive asexual development cycle.

For determination of parasitemia, 50 μl of resuspended parasite culture was mixed with 100 μl of Sybr Green (MilliporeSigma), diluted 1:5000 in FACS Flow (Becton Dickinson, San Diego, CA, USA), and incubated in the dark at 37°C for 15 min. The pellet was washed once in 1 ml FACS Flow and subsequently resuspended in FACS Flow. Measurements were performed on a FACSCalibur (Becton Dickinson), in which 100,000 events were analyzed using Cell Quest v.5.2.1 (BD Biosciences, San Jose, CA, USA).

### Cloning and protein production

PfHsp70-x (*pf3d7_0831700*, Uniprot accession number K7NTP5) constructs were derived from a codon-optimized synthetic DNA fragment from Integrated DNA Technologies (Coralville, IA, USA). PCR was used to amplify DNA sections corresponding to full-length (aa 25–679; excludes export signal peptide) and nucleotide-binding domain (NBD; aa 29–419) PfHsp70-x fragments. Human full-length heat shock cognate protein 70 [Hsc70; heat shock protein family A (*hspa*) member 8, Uniprot accession number P11142] was cloned from sequence-verified cDNA from the Source BioScience Integrated Molecular Analysis of Genomes and Their Expression (I.M.A.G.E.) Consortium. *P. falciparum* PFA0660w (*pf3d7_0113700*, Uniprot accession number Q8I2E1) and PFE0055c (*pf3d7_0501100*, Uniprot accession number Q8I489) constructs were generated using codon-optimized synthetic DNA fragments from Integrated DNA Technologies encoding for protein residues 81–402 and 77–402, respectively. PCR was used to amplify DNA sections corresponding to the J-domains (PFA0660w aa 81–148; PFE0055c aa 77–148). Amplified regions were cloned into a pFloat vector (24) containing an N-terminal His6 tag and a human rhinovirus 3C protease cleave site using Gibson Assembly (New England BioLabs, Ipswich, MA, USA). DNA constructs were sequenced by Eurofins Genomics (Ebersberg, Germany) to confirm correct cloning.

DNA constructs were transformed into *Escherichia coli* Rosetta2(DE3) cells (Merck, Darmstadt, Germany) for protein expression and grown in Luria Bertani medium at 37°C with 50 μg/ml kanamycin and 34 μg/ml chloramphenicol. When the cells reached an optical density at 600 nm of 0.5–0.6, the incubation temperature was reduced to 18°C, and protein expression was initiated by addition of isopropyl β-D-1-thiogalactopyranoside to a final concentration of 250 μM. Protein expression was allowed to proceed for 18 h before harvesting. Cells were harvested by centrifugation and resuspended in ATP lysis buffer (10 mM Na_2_HPO_4_, 2 mM KH_2_PO_4_, 2.7 mM KCl, 500 mM NaCl, 0.01% v/v Triton X-100, 5 mM ATP, 2 mM MgCl_2_ pH 7.4). Sonication was used to lyse resuspended cells, and the resulting lysate was clarified by centrifugation at 30,000 *g*. The clarified lysate was loaded onto a HiTrap Talon metal affinity column (GE Healthcare, Waukesha, WI, USA) preequilibrated with lysis buffer. Proteins were eluted with lysis buffer supplemented with 500 mM imidazole.

Proteins were extensively dialyzed in 3C cleavage buffer (20 mM Na_2_HPO_4_, 150 mM NaCl, 1 mM 1,4-DTT pH 7.4) and incubated with recombinant human rhinovirus 3C protease to cleave the purification tag at 4°C for 16 h. Size exclusion chromatography was performed as the final purification step using Superdex 75 or Superdex 200 columns (GE Healthcare) equilibrated in crystallographic buffer (20 mM HEPES, 50 mM NaCl, 1 mM DTT, pH 7.4) or assay buffer (10 mM HEPES, 100 mM NaCl, 0.5 mM DTT, 2 mM MgCl_2_ pH 8).

Protein purity was assessed at each step by SDS-PAGE, and protein identity was confirmed by time-of-flight mass spectrometry using a Micromass LCT (Waters, Milford, MA, USA). Proteins were concentrated using spin ultrafiltration with concentrations calculated by UV absorption at 280 nm and an extinction coefficient estimated from the amino acid sequence (25).

### Crystallographic structure determination and structure analysis

Diffracting crystals from a sample of 15 mg/ml PfHsp70-x_NBD_ with adenylyl-imidodiphosphate (AMP-PnP) added to a 1:10 molar ratio were obtained at 20°C in a condition containing 24% w/v polyethylene glycol (PEG) 1500 and 20% v/v glycerol using sitting drop vapor diffusion plates (TTP Labtech, Melbourn, United Kingdom) set up using a Mosquito robot. Diffraction data from these crystals were collected at Diamond Light Source beamline I03 at 100 K to a resolution of 1.81 Å. Crystals from a 15 mg/ml sample of PfHsp70-x_NBD_ with a 1:10 molar ratio of ADP were obtained at 20°C in a condition containing 0.1 M 2-ethanesulfonic acid pH 6.0, 5% w/v PEG 3000, and 30% v/v PEG 200. Diffraction data from these crystals were collected at Diamond Light Source beamline I04 at 100 K to a resolution of 1.87 Å. In both cases, the space group was determined to be P 2_1_ 2_1_ 2_1_ with 2 copies of PfHsp70-x_NBD_ per asymmetric unit. Data were processed with xia2 (26) and solved using the automated mode in Phaser (27) with the human Hsp70 NBD domain structure (28) [Protein Data Bank (PDB) ID: 5MKR; *https://www.rcsb.org/*] as the molecular replacement model.

Crystals of PFA0660w_J_ from protein samples at 8.75 mg/ml concentration were obtained at 4°C in a condition containing a 0.12-M mixture of ethylene glycols, 0.1 M Tris-Bicine pH 8.5, 30% w/v PEG monomethyl ether 550, and 30% w/v PEG 20,000. Diffraction data were collected at Diamond Light Source beamline I24 at 100 K to a resolution of 1.39 Å. The space group was determined to be P 1 2_1_ 1 with 2 molecules per asymmetric unit. Data were processed with autoPROC and solved by molecular replacement using Phaser (27) and the J-domain of Sis1 from *Saccharomyces cerevisiae* (29) (PDB ID: 4RWU).

Coot was used for model building alongside iterative refinement with Phenix (30, 31) and Refmac5 (32). Structural models were validated using MolProbity (33). Data collection and refinement statistics can be found in **Table 1**. The crystallographic structures of PfHsp70-x_NBD_:AMP-PnP, PfHsp70-x_NBD_:ADP, and PFA0660w_J_ have been deposited in the Research Collaboratory for Structural Bioinformatics (RCSB) PDB IDs: 6RZQ, 6S02, and 6RZY, respectively. Models were analyzed for cavities and pockets using PockDrug (34). We sought to identify pockets recognizable in all 4 crystallographic models of PfHsp70-x_NBD_ (2 copies of the molecule in each of the AMP-PnP– and ADP-bound structures) and compared these against pockets found in representative structures of human Hsc70_NBD_ and Hsp70_NBD_.

**TABLE 1:** Crystallographic data and refinement statistics

Protein name	PfHsp70-x_NBD_ (AMP-PnP complex)	PfHsp70-xNBD (ADP complex)	PFA0660w_J_
RCSB ID	6RZQ	6S02	6RZY
Beamline	DLS/I03	DLS/I04	DLS/I24
Wavelength (Å)	0.97625	0.9795	1.0389
Space group	P 2_1_ 2_1_ 2_1_	P 2_1_ 2_1_ 2_1_	P 1 2_1_ 1
Unit cell (Å, deg)	80.29 102.71 103.77, 90 90 90	80.12 102.8 103.31, 90 90 90	25.83 54.93 51.44, 90 98.2 90
Resolution range (Å)*^a^*	43.26–1.81 (1.84–1.81)	72.51–1.87 (1.94–1.87)	50.92–1.38 (1.43–1.38)
*R*_merge_ (*I*)*^a^*	0.057 (1.523)	0.068 (1.67)	0.071 (0.600)
*R*_meas_ (*I*)*^a^*	0.062 (1.65)	0.086 (1.82)	0.085 (0.728)
*R*_pim_ (*I*)*^a^*	0.024 (0.63)	0.034 (0.72)	0.049 (0.405)
Completeness (%)*^a^*	99.9 (99.8)	98.78 (94.87)	97.6 (99.4)
Multiplicity*^a^*	6.5 (6.7)	6.3 (6.3)	3.3 (3.1)
*I*/σ*^a^*	12.65 (1.1)	10.33 (1.1)	9.48 (2.04)
CC_1/2_*^a^*	0.99 (0.72)	0.998 (0.582)	0.99 (0.52)
Wilson B factor (Å^2^)*^a^*	38.74	30.99	12.73
Refinement statistics			
*R*_work_ (reflections)	0.192 (70,800)	0.222 (66,212)	0.1647 (28,671)
*R*_free_ (reflections)	0.224 (3923)	0.251 (3386)	0.2027 (1335)
No. of atoms			
Protein	5867	5671*^b^*	1140
Ligands	112	79	—
Water	357	287	124
Mean B factors (Å^2^)			
Protein	54.66	47.02*^b^*	17.1
Ligands	56.50	39.12	—
Water	52.68	40.50	29.3
RMSD from ideal			
Bonds/angles (Å/deg)	0.008/1.49	0.007/0.690	0.009/1.29
MolProbity statistics (33)			
Ramachandran favored (%)	98.65	97.58	100
Ramachandran disallowed (%)	0	0	0
Rotamers favored (%)	97.5	97.3	100
Poor rotamers (%)	0	0.3	0
Clashscore^*c*^	4.42 (97th percentile)	2.4 (99th percentile)	0.88 (99th percentile)
MolProbity score^c^	1.22 (99th percentile)	1.11 (100th percentile)	0.77 (100th percentile)

DLS, diamond light source; RCSB, Research Collaboratory for Structural Biomechanics; *^a^*By xia2 (26). Values in parenthesis correspond to the highest resolution shell. *^b^*Excludes hydrogen atoms. *^c^*100th percentile is the best among structures of comparable resolution; 0th percentile is the worst.

### Biochemical assays

The rate of ATP hydrolysis was measured using a coupled enzymatic reaction in which purine nucleoside phosphorylase enzyme uses released inorganic phosphate to modify the substrate 2-amino-6-mercapto-7-methylpurine riboside (MESG) causing absorption at 360 nm to increase (35). All MESG assays were performed using a ClarioStar microplate reader (BMG Labtech, Ortenberg, Germany). Five micromolars chaperone, supplemented with parasite cochaperones, chaperone inhibitors, or both, as indicated, were incubated with 400 μM MESG and 2 U purine nucleoside phosphorylase enzyme in assay buffer for 30 min at 25°C before the addition of ATP to a final concentration of 1 mM. The absorbance at 360 nm was followed over 2 h and the steady-state reaction rate was calculated. Absorbance values were converted to moles of ATP hydrolyzed using a standard curve derived from variable concentrations of inorganic phosphate samples. Bromo-β-lapachona was sourced from Angene International Ltd. (London, United Kingdom), MKT-077 from MilliporeSigma, and YM-01 from Abcam (Cambridge, United Kingdom).

### NMR assays

NMR experiments were conducted using Bruker Avance III spectrometers (Bruker, Billerica, MA, USA) with cryogenic TCI probeheads at 25°C temperature. Samples consisted of [^13^C] and [^15^N]– or [^15^N]-labeled protein in 20 mM sodium phosphate pH 7.4, 150 mM NaCl, 2 mM DTT, 5% v/v deuterium oxide, and 50 μM 4,4-dimethyl-4-silapentane-1-sulfonic acid buffer. Data were processed with NMRpipe (36) and analyzed with Sparky (37). Sequence-specific resonance assignments were performed using 3-dimensional HN(CO)CACB and HNCACB experiments. Assignments of PFA0660w_J_ and PFE0055c_J_ have been deposited in the Biological Magnetic Resonance Data Bank under accession numbers 27,952 and 27,953, respectively. Peak intensity changes in heteronuclear single quantum coherence experiments were quantified using the following equation, where PC is percentage change in intensity, *PI*_apo_ is peak intensity of labeled protein alone, and *PI*_complex_ is peak intensity in the presence of a binding partner.


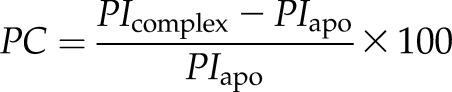


## RESULTS

### PfHsp70-x assists proliferation of ring-stage parasites under heat stress

**Figure 1:**
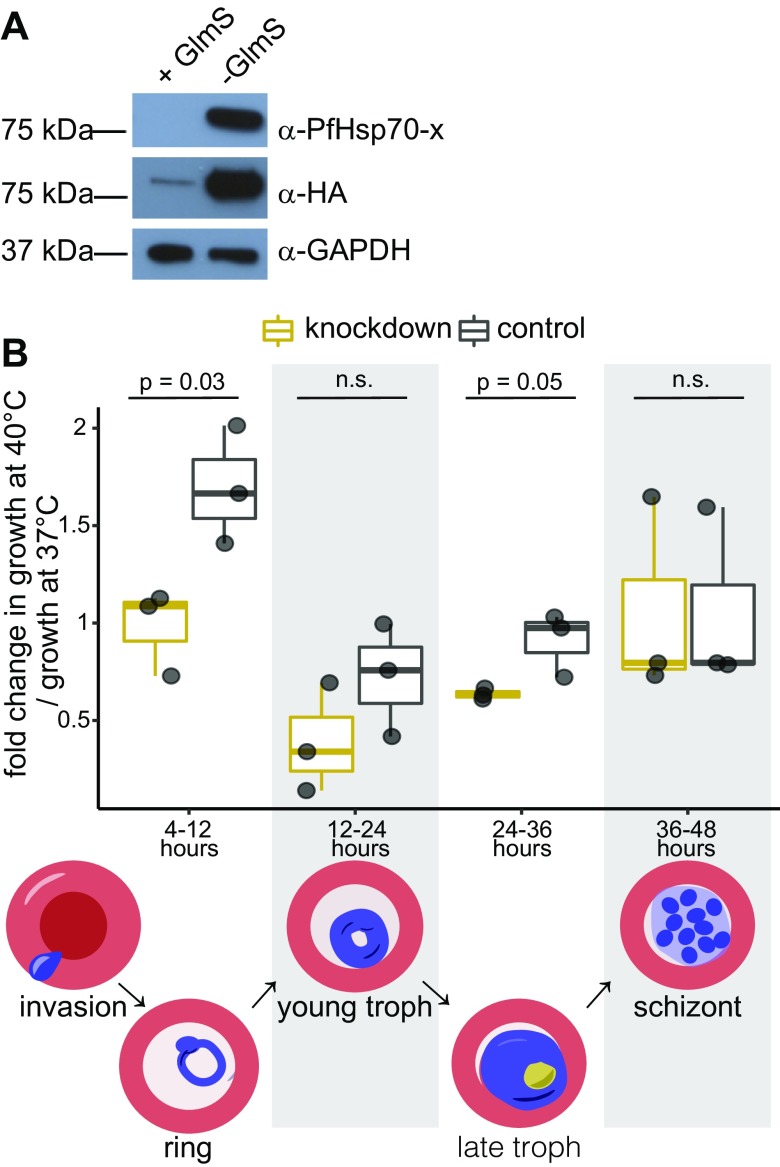
PfHsp70-x support parasite viability under heat shock. *A*) Western blots of lysates from PfHsp70-x_glmS_ parasites cultured with or without glmS, which induces PfHsp70-x knockdown. α-HA antibodies target the HA-tag fused to endogenous PfHsp70-x in this cell line. Glyceraldehyde 3-phosphate dehydrogenase (GAPDH) is used as loading control. *B*) Boxplot showing fold change in parasite growth comparing synchronized cells placed at 40°C for the indicated time interval (4–12, 12–24, 24–36, or 36–48 hpi) *vs.* similar cells grown continuously at 37°C, with or without *glmS*-induced PfHsp70-x knockdown. Means ± sd of 3 biologic replicates are shown, with each point representing the mean of 3 technical replicates. Significance between control and knockdown cells was evaluated by a Wilcoxon rank-sum test. *P* values of null hypotheses are indicated; n.s., no significance. A schematic of parasite life stages is shown below.

Earlier work by Charnaud *et al.* (20) found that the viability of PfHsp70-x knockout parasites under heat shock conditions was similar to that of wild-type cells but noted that differential expression of proteins in this parasite line may compensate for the loss of the exported chaperone. In a parallel study, Cobb *et al.* (21) utilized the *glmS* ribozyme system to deplete PfHsp70-x in a rapid timeframe that does not allow parasite selection but noted no effect on *P. falciparum* growth upon PfHsp70-x knockdown under standard culturing conditions (37°C). In order to evaluate the contribution of PfHsp70-x to parasite stress response, we used the HA-tag and glmS-incorporating parasite line developed by Cobb *et al.* (PfHsp70-x_glmS_) and initially heat shocked unsynchronized parasites to 40°C for 12 h prior to retuning them to standard conditions for the remainder of the parasite 48 h–long life cycle. We found no statistically significant effect on parasite growth upon PfHsp70-x depletion under these conditions; however, we noted that cell growth was highly variable, which impacted the statistical analysis. To overcome this variability in growth we then utilized tightly synchronized parasites and applied heat shocks at different stages of the parasite life cycle (ring, 4–12 hpi; young trophozoite, 12–24 hpi; late trophozoite, 24–36 hpi; schizont 36–48 hpi) and in the absence or presence of GlcN, which activates *hsp70-x* mRNA degradation leading to protein knockdown (**Fig. 1*A***). Parasite growth was compared in all cases with similarly synchronized cells that were not subjected to heat shock.

As shown in Fig. 1*B*, control parasites in which PfHsp70-x was not depleted showed a marked increase in growth (1.7 ± 0.3–fold) when heat shock was applied in the ring stage, compared with similar stage control parasites that were not stressed. This growth enhancement under heat shock is consistent with previous reports of febrile episodes promoting *P. falciparum* development and leading to parasite life cycle synchronization (38, 39). When the heat shock was applied in later parasite stages, this increase in growth was not observed. PfHsp70-x depletion led to an ∼40% reduction in growth of ring-stage parasites under heat shock conditions (*P* = 0.03). Furthermore, young and late trophozoite-stage parasites also demonstrated ∼30% lower growth under heat shock when PfHsp70-x was depleted, although variability in biologic replicates led to this reduction in growth being significant only for late trophozoites (*P* = 0.05). Schizont-stage parasites showed no effect on growth upon PfHsp70-x depletion. We noted that the effect on parasite growth upon PfHsp70-x depletion correlated well with PfHsp70-x expression levels during the intraerythrocytic life cycle (Supplemental Fig. S1) (40) and conclude that PfHsp70-x supports parasite proliferation under heat shock conditions but does so most strongly in early-stage parasites.

### Structural analysis of the PfHsp70-x catalytic domain

We were intrigued by the pattern of PfHsp70-x contribution to parasite virulence (20) and, as seen above, to parasite growth. PfHsp70-x partly supports both these aspects of parasite biology; however, it is not essential for either of them, which suggests that another system complements PfHsp70-x function. We reasoned that such complementation is most likely afforded by human Hsp70 chaperones endogenous to the erythrocyte. Quantitative proteomics have shown that just 2 chaperones, Hsc70 (*HSPA8*) and Hsp70 (*HSPA1A* or *HSPA1B*, which have identical protein sequences), compose 60% and 20%, respectively, of erythrocytic Hsp70s; no other human Hsp70 constitutes more than 10% of this protein class in erythrocytes (41). Thus, we sought to understand the level of similarity between PfHsp70-x and human Hsc70 or Hsp70, and to that end we set out to elucidate the structure of the parasite chaperone.

**Figure 2:**
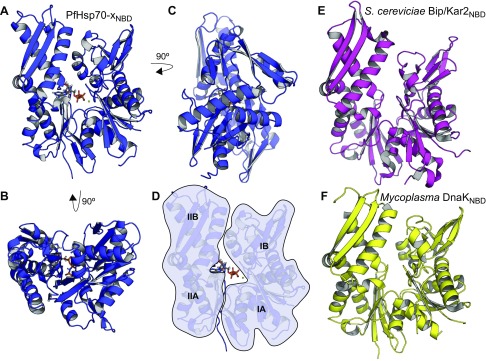
PfHsp70-x_NBD_ adopts a canonical Hsp70 catalytic domain structure. *A*–*C*) Orthogonal views of the PfHsp70-x_NBD_:AMP-PnP crystallographic structure in schematic representation. *D*) An outline of PfHsp70-x_NBD_ structure in the orientation of *A*, where the 2 protein lobes (I and II) and subdomains A and B are indicated. Bound AMP-PnP in the catalytic site is shown as sticks. *E*, *F*) Schematic representations of the yeast binding immunoglobulin protein (Bip/Kar2) Hsp70-type chaperone NBD (PDB ID 3QFP) (*E*) and the *Mycoplasma genitalium* DnaK_NBD_ (PDB ID 5OBY) (*F*) in the same orientation as PfHsp70-x_NBD_ in *A*. The structural similarity (Cα RMSD) between these catalytic domains and PfHsp70-x_NBD_:AMP-PnP is 0.7 and 0.55 Å, respectively.

Hsp70 chaperones comprise an NBD, which hydrolyzes ATP to drive the protein refolding cycle, and a bipartite substrate-binding domain (SBD) composed of a hydrophobic β-sandwich (SBD-β) and an α-helical “lid” (SBD-α) that trap between them the misfolded target (42). We resolved the 1.81 and 1.87 Å resolution crystallographic structures (data collection and refinement statistics shown in Table 1) of the PfHsp70-x_NBD_ in complex with a nonhydrolysable ATP analog (AMP-PnP) and ADP, respectively. PfHsp70-x_NBD_ crystals included 2 copies of the protein per asymmetric unit with nearly identical structures [Cα atom root mean square deviation (RMSD) of 0.26 and 0.32 Å for the AMP-PnP and ADP crystal forms, respectively]. The overall structure of PfHsp70-x_NBD_ (**Fig. 2*A–C***) was similar to that of other Hsp70 NBDs (43), comprising 2 lobes (I and II), each consisting of 2 subdomains (A and B), with a channel between them leading to the nucleotide-binding site (Fig. 2*D*). For example, comparison of the PfHsp70-x_NBD_ structure and that of prototypical eukaryotic or bacterial Hsp70 catalytic domains yielded Cα RMSDs of 0.5–0.7 Å (Fig. 2*E, F*). ATP binding and hydrolysis drive the structural rearrangement of Hsp70 NBD subdomains, as shown in previous structural studies of this chaperone class (44). However, we found that both the PfHsp70-x_NBD_ AMP-PnP and ADP complex states captured in crystals were highly similar (mean Cα RMSD of 0.37 Å) and corresponded most closely to hydrolyzed ATP– and ADP-bound states of previously resolved Hsp70 NBDs.

### PfHsp70-x is highly similar to the human Hsc70 and Hsp70 chaperones

**Figure 3:**
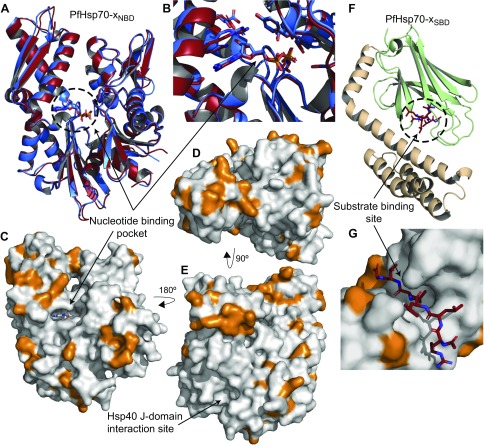
PfHsp70-x is structurally similar to human Hsp70 chaperones. *A*) Overlay of the PfHsp70-x_NBD_:AMP-PnP crystallographic structure (blue) and human Hsc70_NBD_:ADP (red; PDB ID 4H5T) in schematic representations. *B*) Magnification of the nucleotide-binding pocket and residues surrounding the nucleotide. *C*–*E*) Views of PfHsp70-x in surface representation with amino acids differing between the parasite chaperone and human Hsc70 or Hsp70 (sequence alignment in Supplemental Fig. S2) colored in orange. The nucleotide-binding pocket and Hsp40 J-domain interaction sites are indicated. *F*) Homology model of PfHsp70-x_SBD_ based on the crystallographic structure of the human Hsp70_SBD_ (PDB ID 4PO2). The SBD-α and SBD-β subdomains are colored beige and green, respectively. A model substrate peptide is shown in stick representation (red) complexed to SBD-β. *G*) Surface representation of the PfHsp70-xSBD-β substrate-binding cleft with amino acids differing between the parasite chaperone and human Hsc70 or Hsp70 colored in orange. Model substrate peptide shown as in *F*.

Comparison of human Hsc70_NBD_ in the hydrolyzed ATP–bound state (45) (PDB ID 4H5T) with PfHsp70-x_NBD_ revealed a nearly identical structure with Cα RMSD of ∼0.4 Å (**Fig. 3*A***); comparison of Hsp70_NBD_ (46) (PDB ID 4J8F) and PfHsp70-x_NBD_ yielded similar results. These exceptional levels of structural similarity are consistent with the very high degree of sequence homology seen between PfHsp70-x_NBD_ and Hsc70_NBD_ or Hsp70_NBD_ (over 70% aa identity in both cases; Supplemental Fig. S2). Mapping of amino acid differences between these NBDs showed that these do not form a single, large surface, which might have suggested differences in binding of protein partners, but rather that they are dispersed throughout the protein surface (Fig. 3*C–E*). We noted, in particular, that the NBD subdomain IA surface that forms the canonical interface for Hsp70 recognition of stimulatory Hsp40 cochaperones is identical across PfHsp70-x_NBD_ and Hsc70_NBD_ or Hsp70_NBD_ (Fig. 3*E*), as is the catalytic site of ATP hydrolysis (Fig. 3*B* and Supplemental Fig. S2).

Despite repeated attempts, we were unable to obtain diffracting crystals of full-length PfHsp70-x or of recombinant fragments corresponding to the SBD of this protein. Similarly, the SBD of human Hsc70 has not been structurally resolved previously, whereas the structure of Hsp70_SBD_ has been determined (47) (PDB ID 4PO2). Thus, we used homology modeling to construct a representative structure of PfHsp70-x_SBD_ (Fig. 3*F*), which served as a basis for comparison. Sequence similarity between the parasite and human chaperone SBDs is slightly lower compared with the NBDs though remaining at over 60% amino acid identity, with most changes localized to the lid (SBD-α) subdomain (Supplemental Fig. S2). Crucially, none of the amino acid residues involved in direct substrate binding is substantially different between PfHsp70-x_SBD_ and the 2 human chaperones (Fig. 3*G*).

We surmise that the structural and sequence similarities between PfHsp70-x and human Hsc70 or Hsp70 suggest that all these chaperones have the potential to associate with the same stimulatory cochaperones and substrate proteins. In this manner, PfHsp70-x and Hsc70 or Hsp70 may be functionally interoperable for parasite viability and cytoadherence.

### Parasite cochaperones stimulate PfHsp70-x and Hsc70 activity

Hsp40 cochaperones stimulate Hsp70 activity and expand the repertoire of substrate proteins that may be operated on by the chaperones (48, 49). Cochaperone specificity is, therefore, key for understanding Hsp70 function. Previous studies showed that PFA0660w, a parasite cochaperone that localizes with PfHsp70-x in J-dots in the erythrocyte (16), preferentially stimulates PfHsp70-x activity compared with human Hsp70 (19). At least one other parasite cochaperone, PFE0055c, is also known to locate in J-dots (16). Proximity-ligation assays suggested that PFA0660w and PFE0055c participate in complexes that include PfHsp70-x in the infected erythrocyte cytosol (16). PFA0660w and PFE0055c differ in their expression profiles (40), with PFE0055c levels being constant throughout the parasite intraerythrocytic life cycle. In contrast, PFA0660w levels peak at the ring stage (40), when PfHsp70-x expression levels and effect on parasite viability under stress are at maximum. Given the PfHsp70-x, Hsc70, and Hsp70 interoperability suggested by the structural analysis above, we set out to assess the direct interaction of the parasite and human chaperones with PFA0660w and PFE0055c.

**Figure 4:**
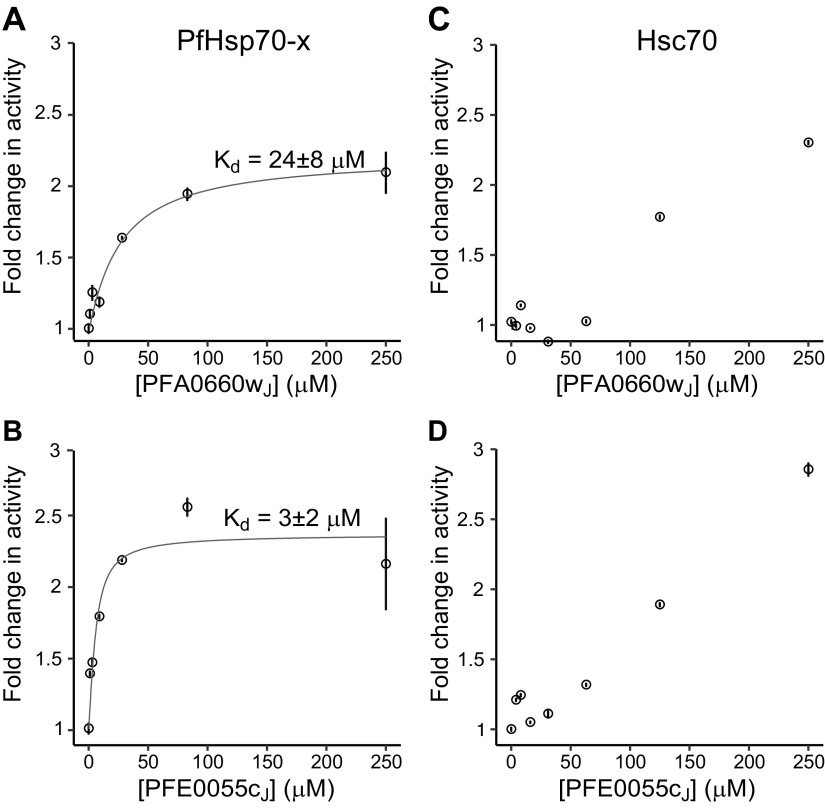
Stimulation of Hsp70 activity by parasite cochaperones. Shown here are fold changes in ATP hydrolysis activities of PfHsp70-x (*A*, *B*) or human Hsc70 (*C*, *D*) upon titration of recombinant parasite PFA0660w (*A*, *C*) or PFE0055c (*B*, *D*) Hsp40 J-domains. ATP hydrolysis activities were measured as initial reaction rates using 5 μM chaperone concentrations and scaled to activities in the absence of cochaperones. Data points correspond to means ± sd from triplicate experiments. PfHsp70-x activities were fitted to ideal single-site binding models (solid lines) to extract the indicated apparent dissociation constants (*K*_d_) for the interaction of PfHsp70-x with cochaperones.

We initially compared the ability of recombinant J-domains from PFA0660w and PFE0055c to stimulate chaperone activity, as measured by ATP hydrolysis. Hsp40 J-domains are believed to be sufficient for such stimulation (49). Consistent with previous studies using full-length PFA0660w, the J-domain of this protein (PFA0660w_J_) stimulated PfHsp70-x activity by ∼2-fold (**Fig. 4*A***). A similar level of stimulation was observed when using the PFE0055c J-domain (PFE0055c_J_; Fig. 4*B*). The apparent interaction affinities (dissociation constants) between PfHsp70-x and these J-domains were estimated to be in the low μM range (PFA0660w_J_ ∼25 μM; PFE0055c_J_ ∼3 μM) by titrating the amount of cochaperone in the ATP hydrolysis assays, with maximal stimulation of activity occurring at ∼10–20:1 cochaperone:chaperone molar ratios. We further noted that PFA0660w_J_ and PFE0055c_J_ are substantially less efficient at stimulating Hsc70 ATP hydrolysis; although the human chaperone activity could be induced by high parasite cochaperone concentrations, we found no indication of activity plateauing even at molar ratios as high as 50:1 (Fig. 4*C, D*), which suggests very weak binding of Hsc70 with PFA0660w_J_ and PFE0055c_J_ . We surmise that malarial exported Hsp40 cochaperones can stimulate the activities of both parasite PfHsp70-x and human Hsc70, albeit substantially less efficiently in the latter case.

### PFA0660w associates with PfHsp70-x and Hsc70 into canonical chaperone-cochaperone complexes

We sought to investigate the structural basis of PfHsp70-x and Hsc70 stimulation by parasite cochaperones, focusing particularly on PFA0660w whose expression coincides with large-scale erythrocyte remodeling. To that end, we resolved the crystallographic structure of PFA0660w_J_ at 1.38 Å resolution (Table 1 and Supplemental Fig. S3) and assigned the NMR spectra of PFA0660w_J_ in a sequence-specific manner. This allowed us to study the physical interaction of PFA0660w_J_ with PfHsp70-x or Hsc70 at amino acid–level resolution (Supplemental Fig. S4). As anticipated from the chaperone stimulation assays above, both PfHsp70-x and Hsc70 interacted directly with PFA0660w_J_, as evidenced by changes in the intensity of [^15^N]-labeled PFA0660w_J_ NMR peaks corresponding to specific amino acids upon titration of this protein with unlabeled PfHsp70-x or Hsc70 (Supplemental Fig. S4*C*). Furthermore, we noted that PfHsp70-x produced larger changes in NMR peak intensity compared with Hsc70, suggesting that the PfHsp70-x–PFA0660w_J_ interaction is stronger than that between PFA0660w_J_ and the human chaperone, which is consistent with the biochemical assays above (Fig. 4).

**Figure 5:**
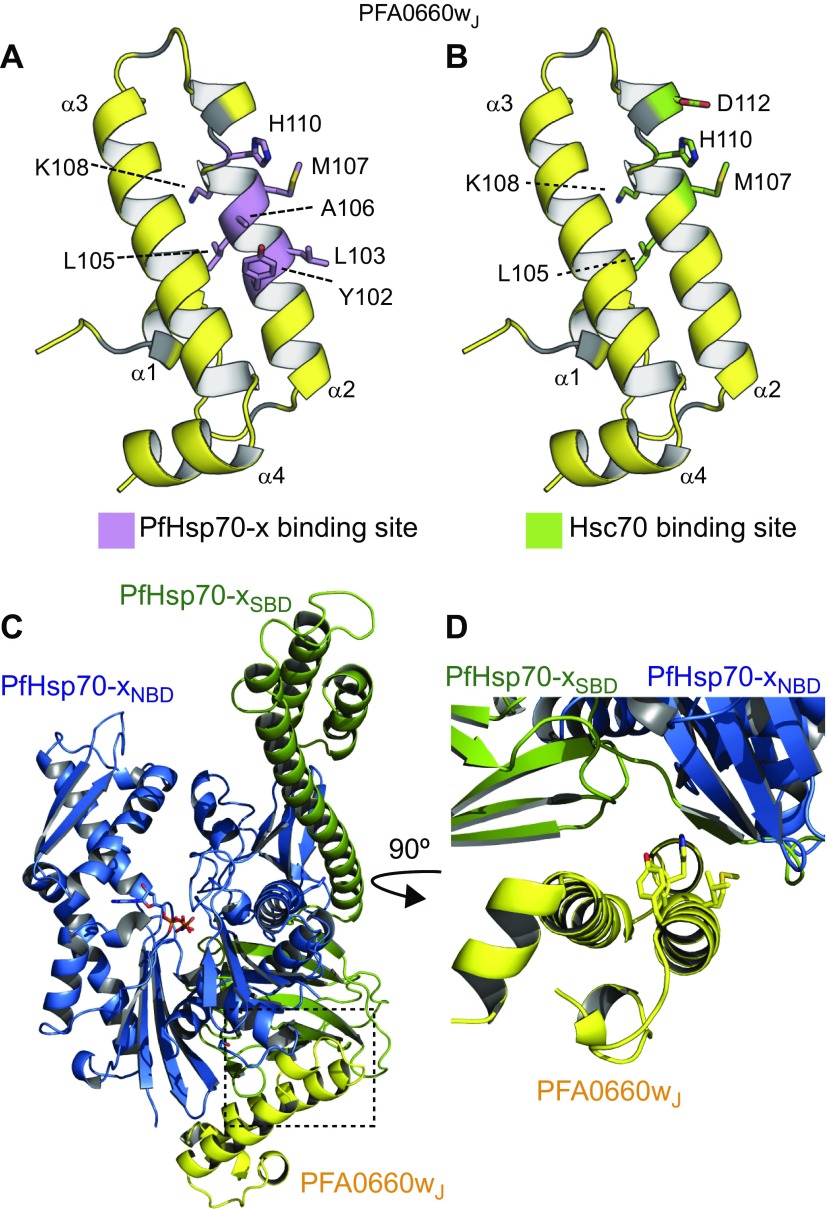
Hsp70 chaperones form a canonical complex with parasite cochaperones. *A*, *B*) Schematic representations of the PFA0660w_J_ crystallographic structure with residues determined by solution NMR assays to interact with PfHsp70-x (*A*) or human Hsc70 (*B*) named and shown as sticks. The secondary structure elements of PFA0660w_J_ are indicated. *C*, *D*) Orthogonal view (*C*) and magnification (*D*) of the PfHsp70-x–PFA0660w_J_ complex modeled on the basis of the DnaK-DnaJ_J_ complex structure (PDB ID 5NRO). Compared with that complex, the DnaK_NBD_ and DnaJ_J_ domains were replaced by the PfHsp70-x_NBD_ and PFA0660w_J_ structures, respectively. PFA0660w_J_ residues involved in PfHsp70-x binding as determined by solution NMR assays are shown as sticks.

To evaluate whether these interactions are compatible with the formation of canonical chaperone-cochaperone complexes, we mapped the PFA0660w_J_ amino acids showing the largest changes in NMR assays upon complex formation to the cochaperone domain structure; these amino acids are expected to define the direct protein binding interface. As seen in **Fig. 5*A*, B**, both the PfHsp70-x and Hsc70 interactions primarily involved residues of the PFA0660w_J_ α-helix 2 (α2) forming a continuous binding surface toward the C terminus of that helix. Notably, these residues included PFA0660w H110, which is part of a conserved HPD motif found in Hsp40 cochaperones that is necessary for stimulation of Hsp70 activity (50). Modeling of the PfHsp70-x–PFA0660w complex based on the recently published crystallographic structure of full-length DnaK bound to DnaJ_J_ (PDB ID 5NRO) (51) revealed that the PFA0660w_J_ α2 interaction interface highlighted in NMR assays agrees well with amino acids anchoring the cochaperone to the chaperone NBD, thereby stimulating chaperone activity (Fig. 5*C*, *D*). These results suggest that PFA0660w_J_ can form canonical chaperone-cochaperone complexes with both parasite PfHsp70-x and human Hsc70; these complexes are likely at the heart of chaperone stimulation by PFA0660w demonstrated earlier.

### Small-molecule inhibition of PfHsp70-x

**Figure 6:**
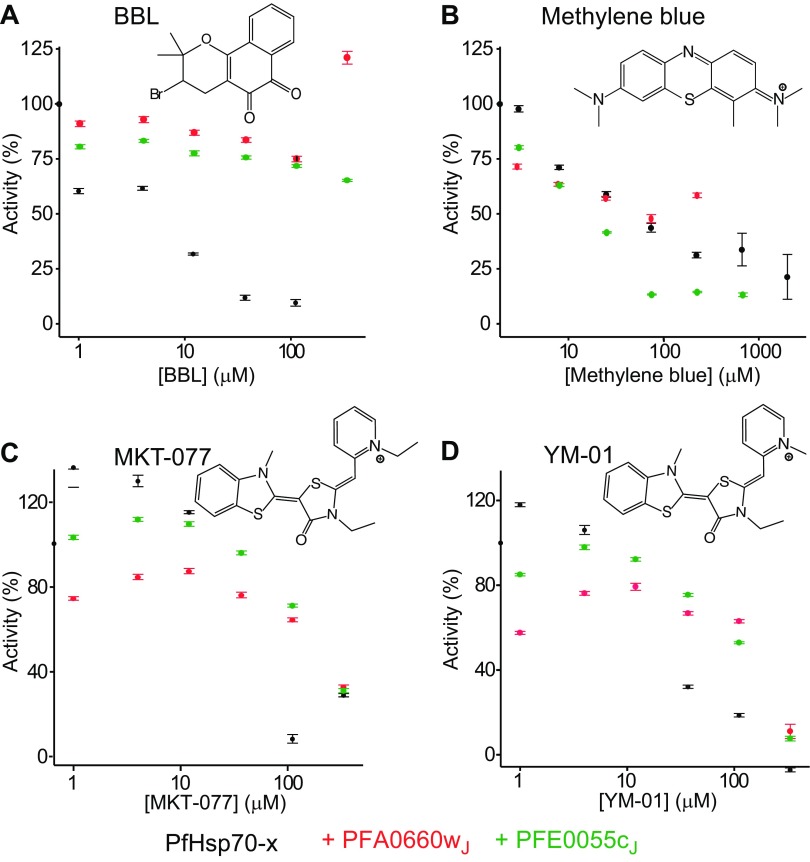
Small-molecule inhibition of PfHsp70-x activity. Shown here are changes in PfHsp70-x ATP hydrolysis activity upon titration of BBL (*A*), methylene blue (*B*), MKT-077 (*C*), and YM-01 (*D*) inhibitors. Assays were performed with 5 μM PfHsp70-x alone (black) or in the presence of 75 μM PFA0660w_J_ (red) or PFE0055c_J_ (green) cochaperones. Data points correspond to means ± sd from triplicate experiments. Activities were scaled relative to initial activities in the absence of inhibitor molecules. The chemical structure of inhibitors is shown.

Given the high degree of structural similarity between PfHsp70-x and human Hsc70 or Hsp70, and in their interactions with PFA0660w, we wondered whether small molecules might be able to specifically inhibit the parasite system, thereby offering a chemical avenue for teasing out the contributions of different chaperones to parasite viability. In particular, bromo-β-lapachona (BBL) has been suggested to specifically inhibit the ATPase activity of PfHsp70-x but not that of the parasite intracytosolic PfHsp70-1 or of human Hsp70 (52). Furthermore, BBL was shown to attenuate PfHsp70-x activity in the presence of the promiscuous human Hsp40 cochaperone heat shock protein J-domain 1a (Hsj1a) and the parasite intracytosolic *P. falciparum* Hsp40. Consistent with these previous results, BBL inhibited PfHsp70-x in our ATPase assays with low micromolar apparent half-maximal inhibitory concentration (**Fig. 6*A***). However, BBL did not substantially affect PfHsp70-x activity when the chaperone was stimulated by its cognate cochaperones PFA0660w_J_ or PFE0055c_J_, which reflects the more likely case inside the infected erythrocyte.

We then turned to known inhibitors of Hsp70 chaperones that have been reported to exhibit antimalarial activity. Methylene blue, a phenothiazine dye, has been shown to inhibit heat-induced human Hsp70s through oxidation of an exposed cysteine in the ATPase domain (53). The dye has also been historically used as an antimalarial, and a number of recent clinical trials found it to be safe and effective against *P. falciparum* infections (54–56). ATPase assays revealed that methylene blue inhibits both basal and stimulated PfHsp70-x activity (Fig. 6*B*), but only at relatively high concentrations of the dye (tens of micromolars half-maximal inhibitory concentration). We further assessed MKT-077, a rhodacyanine dye, and its derivative, YM-01, which exhibit antitumor activity through inhibition of Hsp70s by binding to a conserved allosteric pocket near the catalytic site (Supplemental Fig. S2) (57–59). MKT-077 was found in a recent study to be 14 times more toxic to *P. falciparum* than to human cells (60). Our assays suggest that MKT-077 and YM-01 exhibit minimal PfHsp70-x inhibition unless in concentrations of over 100 μM (Fig. 6*C, D*). Thus, we conclude that both potentially specific (BBL) and broad-spectrum (methylene blue, MKT-077, YM-01) Hsp70 inhibitors show relatively little potency against PfHsp70-x, especially when this chaperone is stimulated by its *in vivo* partners.

## DISCUSSION

*P. falciparum* development occurs in a highly challenging environment for protein homeostasis, a key requirement for life to maintain protein structure and thereby function. The parasite is faced with elevated host temperature every ∼48 h, because of fever, and high levels of free radical species generated by heme from hemoglobin digestion. *P. falciparum* responds to these sources of stress through a dedicated mechanism for heme detoxification (13), by co-opting host components that assist protein folding (61, 62), and by expressing a large complement of molecular chaperones (11, 12). The importance of these mechanisms for parasite viability is illustrated by the number of antimalarial therapeutics that act by interfering with protein homeostasis directly or indirectly. For example, chloroquine interferes with the heme detoxification process (63), leading to increased free radicals that damage biologic macromolecules, whereas artemisinin is converted to free radical upon activation that promiscuously attacks parasite proteins (64). Thus, interfering with parasite housekeeping mechanisms is a proven avenue for antimalarial drugs. Because resistance to many antimalarial drugs has spread throughout malaria endemic countries (65–68), it is now more important than ever to expand our arsenal of antimalarials and to discover novel molecular targets (69).

Targeting protein chaperones has been the focus of recent therapeutics against a range of diseases. Interest in Hsp70s, in particular, is high because they lie at the center of the chaperone network and are key for cellular upkeep and survival; for example, Hsp70s and the interactions of Hsp70s with their cognate Hsp40s have been targeted in cancer treatment and neurodegenerative diseases (70–72). Thus, a key question is whether parasite chaperones might similarly provide therapeutic possibilities. To answer this question, we need to address the role of these chaperones in parasite survival and virulence and to understand the potential for selective targeting of *P. falciparum* enzymes.

Our work here demonstrated for the first time a direct contribution of PfHsp70-x to parasite survival, in the form of ∼40% reduction in parasite growth upon chaperone depletion (Fig. 1) under heat shock conditions. This PfHsp70-x effect on growth depends strongly on timing the heat shock to coincide with early stages of the parasite intraerythrocytic life cycle. Previous PfHsp70-x studies did not note this chaperone contribution either because no parasite stress was applied (21) or, likely, because of changes in parasite protein expression compensating for the loss of PfHsp70-x (20). Key for our observations was the rapid depletion of PfHsp70-x through the glmS system, thereby avoiding compensation mechanisms, and tight synchronization of parasites, which allowed us to note the dependence of the PfHsp70-x growth effect on heat shock timing.

To judge whether such a finely timed effect is biologically significant, it is worth considering the physiologic context of PfHsp70-x function. Similar to other pathogens, *P. falciparum* has coevolved with the human host and is highly tuned to its responses (73), thereby maximizing parasite fitness. High fevers are frequently observed in *P. falciparum* malaria and are a clear sign of life-threatening disease, mainly induced by host cell rupture during the 48-h parasite life cycle and release of factors inducing inflammation. Our work suggests that *P. falciparum* anticipates elevated host temperatures coinciding with the destruction of host cells and early parasite development and that it has developed mechanisms to counter, and even benefit from, this temporary heat shock. Previous studies noted a similar effect, whereby febrile episodes promoted *P. falciparum* development (38) and caused synchronization of the parasite life cycle in cultures (39). As shown by protein depletion experiments (Fig. 1), PfHsp70-x provides one of the parasite fever-adjustment mechanisms active at the beginning of the intraerythrocytic life cycle. Hsp70-x is found exclusively in parasites in the *Laverania* subgenus (16), which export a larger complement of proteins to their host cell compared with other *Plasmodia* (3), including virulence factors such as PfEMP1 (8). This enhanced complement of exported proteins places additional pressure on the mechanisms for host homeostasis, such as existing erythrocytic chaperones. Thus, PfHsp70-x may support parasite growth at elevated temperatures by simply providing additional chaperone capacity in the infected cell. However, we cannot exclude the alternatively possibility that PfHsp70-x is particularly, or even solely, responsible for assisting specific parasite proteins in maintaining their structure and function. In either case, we conclude that PfHsp70-x is a key part of the parasite’s evolution-driven efforts to adapt to host conditions and maximize its chances for survival, which include endothelial adhesion supported by PfHsp70-x (20) and adjustment to febrile episodes. As such, we postulate that PfHsp70-x is a high-value target for efforts aiming to elucidate how the parasite adjusts to its host.

Can PfHsp70-x be selectively targeted for inhibition in the presence of human Hsp70 chaperones endogenous to the erythrocyte? Structural analysis initially argued against this, because the NBDs and SBDs of PfHsp70-x and human Hsc70 or Hsp70 are highly similar (Fig. 3), and both parasite and human chaperones can form productive complexes with the parasite cochaperones that influence substrate specificity (Fig. 5). However, crystallographic structures offer only a still snapshot of protein conformation, because they do not reveal how conformationally flexible these proteins might be. It is well understood that protein conformational flexibility, which can even be driven by intrinsically disordered regions not resolved by crystallography (74), leads to differences in binding to small molecules, allosteric modulators, and other proteins (75, 76). Indeed, our results show that despite the high level of similarity, PfHsp70-x binds much more strongly to cognate parasite cochaperones than do human chaperones (Fig. 4), whereas known inhibitors of human Hsc70 or Hsp70 have much less effect on PfHsp70-x than might be expected (Fig. 6). These results suggest that PfHsp70-x and human Hsc70 or Hsp70 differ in their functionally important conformational space.

**Figure 7:**
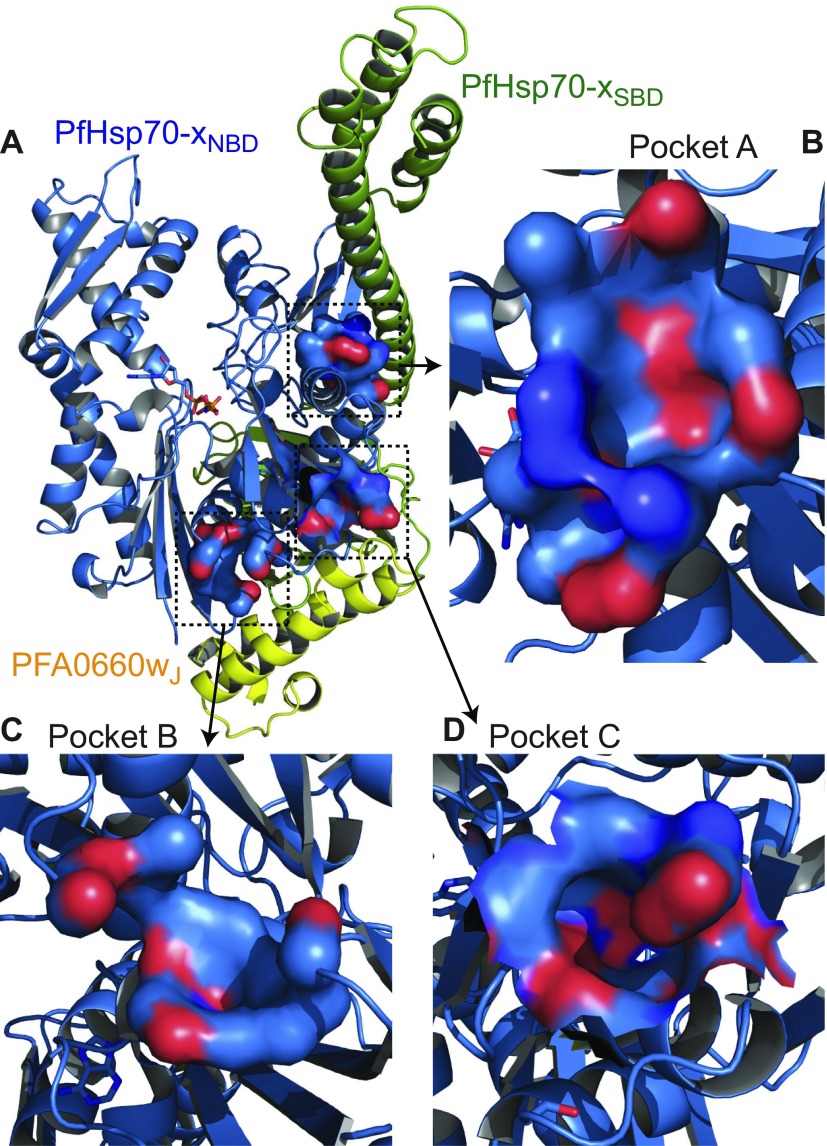
Putative drug-binding pockets on PfHsp70-x. *A*) The PfHsp70-x–PFA0660w complex of Fig. 5*C* in schematic representation with predicted pockets for small-molecule binding shown as solvent-accessible surfaces. Surface atom types are denoted by color: red, oxygen; blue, carbon, dark blue, nitrogen. *B*–*D*) Enlarged views of pocket A (*B*), pocket B (*C*), and pocket C (*D*). Pockets predicted by PockDrug (34) and selected to include amino acids that vary between PfHsp70-x and human Hsc70 or Hsp70.

How could PfHsp70-x be selectively targeted? Analysis of the PfHsp70-x_NBD_ structure for pockets that may provide binding sites to small molecules offers a number of possibilities. In **Fig. 7**, we highlight 3 pockets in particular as being of potentially high interest. Pocket A (Fig. 7*B*) faces the PfHsp70-x_SBD_ (Fig. 7*A*) and, thus, may provide means to block the association of NBDs and SBDs crucial for chaperone function (77). In contrast, pockets B and C (Fig. 7*C, D*) are adjacent to the chaperone-cochaperone binding interface (Fig. 7*A*) and may offer opportunities for allosteric control of PfHsp70-x stimulation by its cognate cochaperones. Importantly, all 3 sites were judged as “drug-able” by the PockDrug structure-based prediction server (34), with probabilities of 50, 100, and 82% for pockets A, B, and C, respectively, while also featuring at least one substantive amino acid difference between PfHsp70-x and human Hsc70 or Hsp70 (pocket A: K155→T; pocket B: S417→E; pocket C: E33→G/A, V34→P/A, and R51→Q). Although these binding predictions remain to be validated by experiments, we postulate that selective targeting of PfHsp70-x function and stimulation is possible.

In summary, we present here a functional analysis of PfHsp70-x that supports a role for this protein in helping the parasite adjust to host febrile episodes. Structural analysis of PfHsp70-x shows a high degree of similarity between this chaperone and human components endogenous to the erythrocyte; however, parasite and human chaperones are shown to have distinct interaction properties with stimulatory cochaperones and inhibitors. Thus, an opportunity exists to identify small molecules that would specific inhibit PfHsp70-x, thereby allowing us to disentangle the roles of this human and parasite chaperones in *P. falciparum* cell biology.

## Supplementary Material

This article includes supplemental data. Please visit *http://www.fasebj.org* to obtain this information.

## Abbreviations

AMP-PnP: adenylyl-imidodiphosphate
BBL: bromo-β-lapachona
GlcN: glucosamine
glmS: GlcN-6-phosphate riboswitch
HA: influenza hemagglutinin
hpi: hours postinfection
HRP: horseradish peroxidase
Hsc70: heat shock cognate protein 70
Hsp: heat shock protein
HSPA: heat shock protein family A
ID: identifier
MESG: 2-amino-6-mercapto-7-methylpurine riboside
NBD: nucleotide-binding domain
PDB: Protein Data Bank
PEG: polyethylene glycol
PfEMP1: *P. falciparum* erythrocyte membrane protein 1
PfHsp70-x: *P. falciparum* Hsp70-x
RMSD: root mean square deviation
SBD: substrate-binding domain
