# Supplementary material for: The Plasmodium falciparum Hsp70-x chaperone assists the heat stress response of the malaria parasite
Source: FASEB J. 2019 Nov 14;33(12):14611–24. doi: 10.1096/fj.201901741R (PMC6894070; doi:10.1096/fj.201901741R)
Supplement: Supplementary file 1 [file fj.201901741R.sf1.pdf]

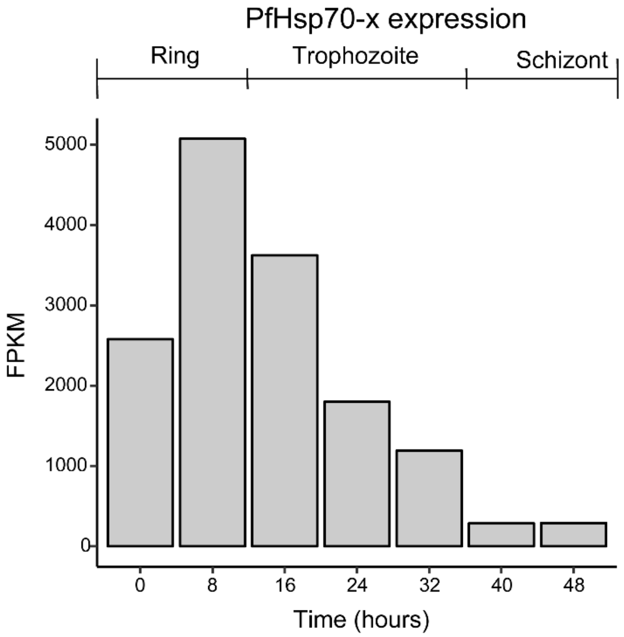

Supplemental Fig. 1: PfHsp70-x expression levels during intra-erythrocytic parasite development. Shown here are *P. falciparum hsp-70x* transcript levels of fragments per kilobase of exon model per million of mapped reads (FPKM) against hours of parasite development post erythrocyte invasion. Data extracted from [40].
