# Supplementary material for: The Plasmodium falciparum Hsp70-x chaperone assists the heat stress response of the malaria parasite
Source: FASEB J. 2019 Nov 14;33(12):14611–24. doi: 10.1096/fj.201901741R (PMC6894070; doi:10.1096/fj.201901741R)
Supplement: Supplementary file 2 [file fj.201901741R.sf2.pdf]

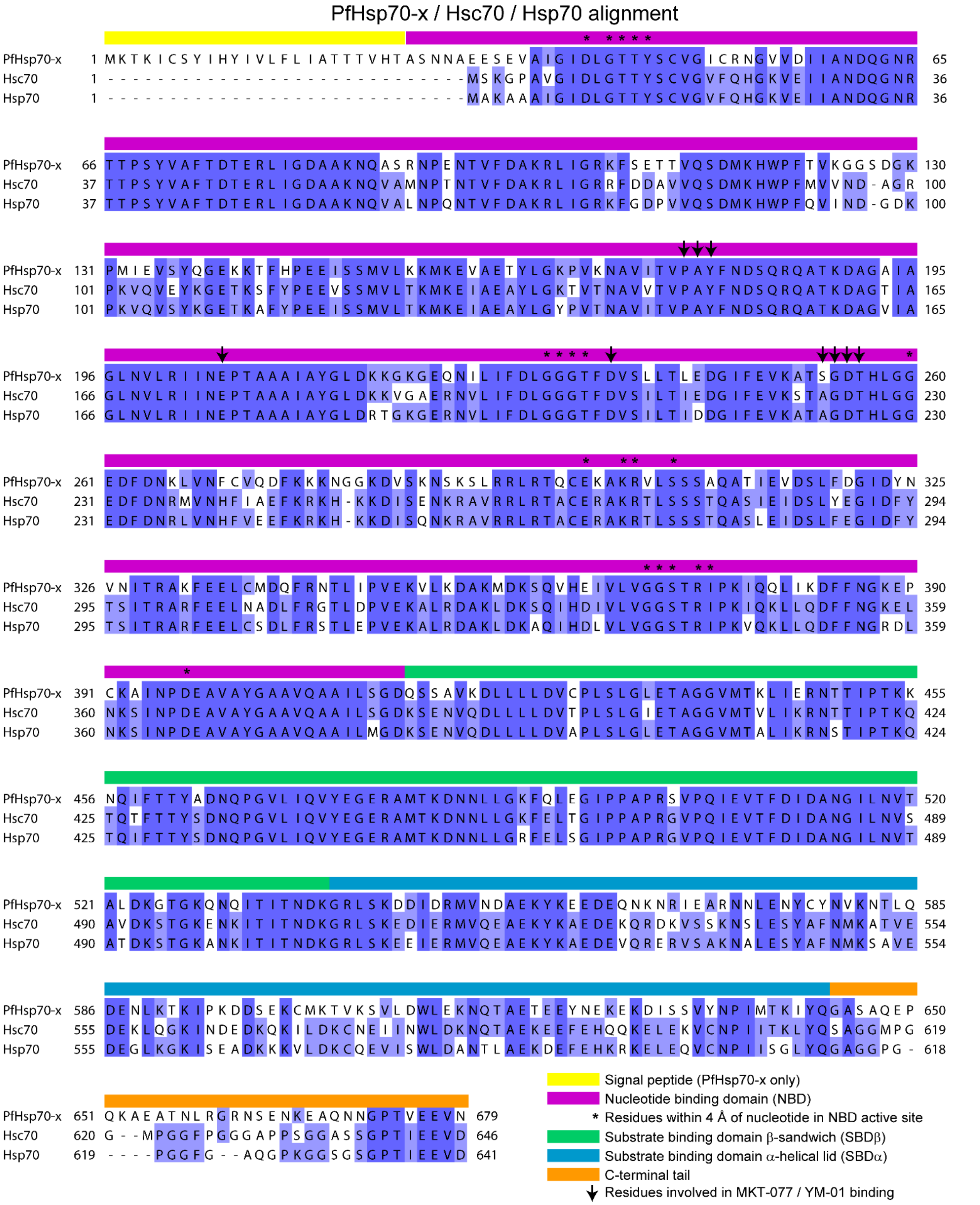

Supplemental Fig. 2: Alignment of PfHsp70-x with human erythrocytic Hsp70 chaperones. Shown here is the amino acid sequence alignment of PfHsp70-x, and human Hsc70 and Hsp70 chaperones. The extent of Hsp70 domains in the sequence is indicated by colored spans. Asterisks denote amino acid residues located within 4 Å of the nucleotide binding site. Arrows denote residues involved in MKT-077 and YM-01 binding [57-59].
