# Supplementary material for: The Plasmodium falciparum Hsp70-x chaperone assists the heat stress response of the malaria parasite
Source: FASEB J. 2019 Nov 14;33(12):14611–24. doi: 10.1096/fj.201901741R (PMC6894070; doi:10.1096/fj.201901741R)
Supplement: Supplementary file 3 [file fj.201901741R.sf3.pdf]

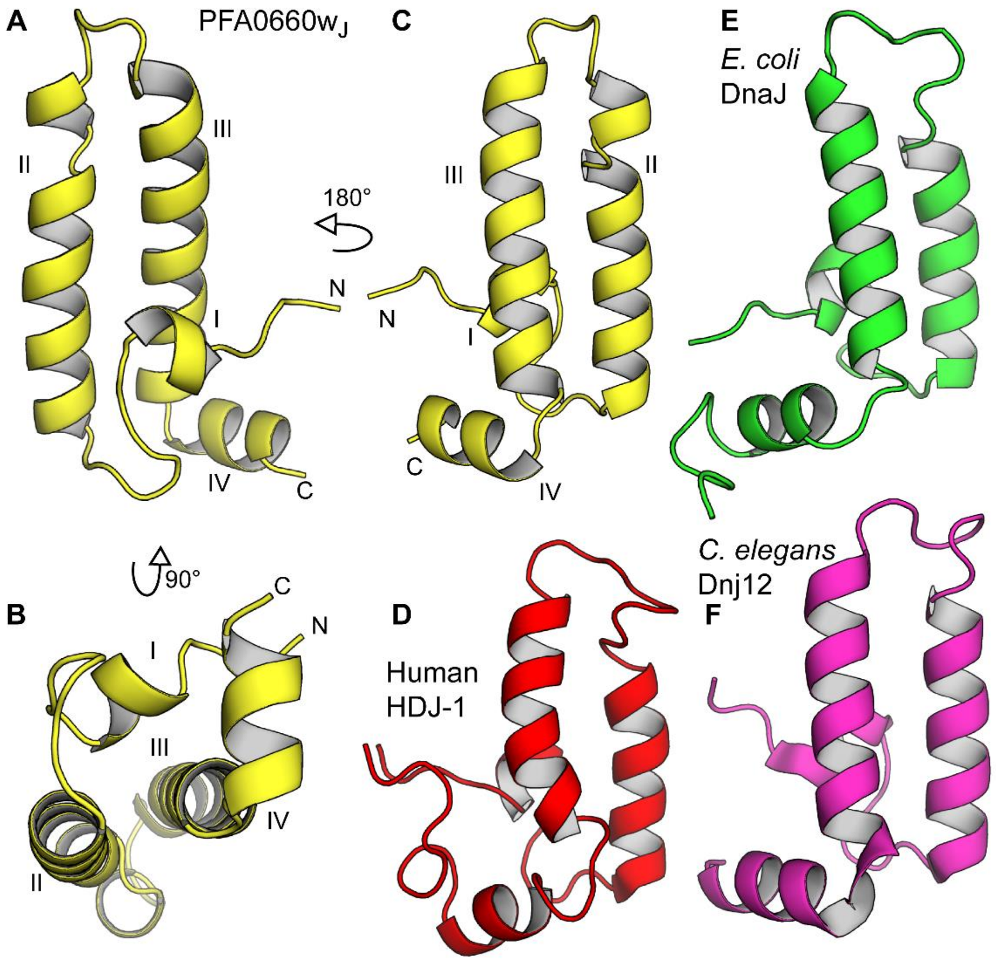

Supplemental Fig. 3: PFA0660w$_J$ domain structure. (A-C) Orthogonal views of the PFA0660w$_J$ crystallographic structure in schematic representations. (D-F) Schematic representations of the J-domains from the human HDJ-1 (D, PDB ID 1HDJ), *E. coli* DnaJ (E, PDB ID 1XBL) and *Caenorhabditis elegans* Dnj12 (F, PDB ID 2OCH) Hsp40 co-chaperones in the same orientation as PFA0660w$_J$ in panel (A).
