# Supplementary material for: The Plasmodium falciparum Hsp70-x chaperone assists the heat stress response of the malaria parasite
Source: FASEB J. 2019 Nov 14;33(12):14611–24. doi: 10.1096/fj.201901741R (PMC6894070; doi:10.1096/fj.201901741R)
Supplement: Supplementary file 4 [file fj.201901741R.sf4.pdf]

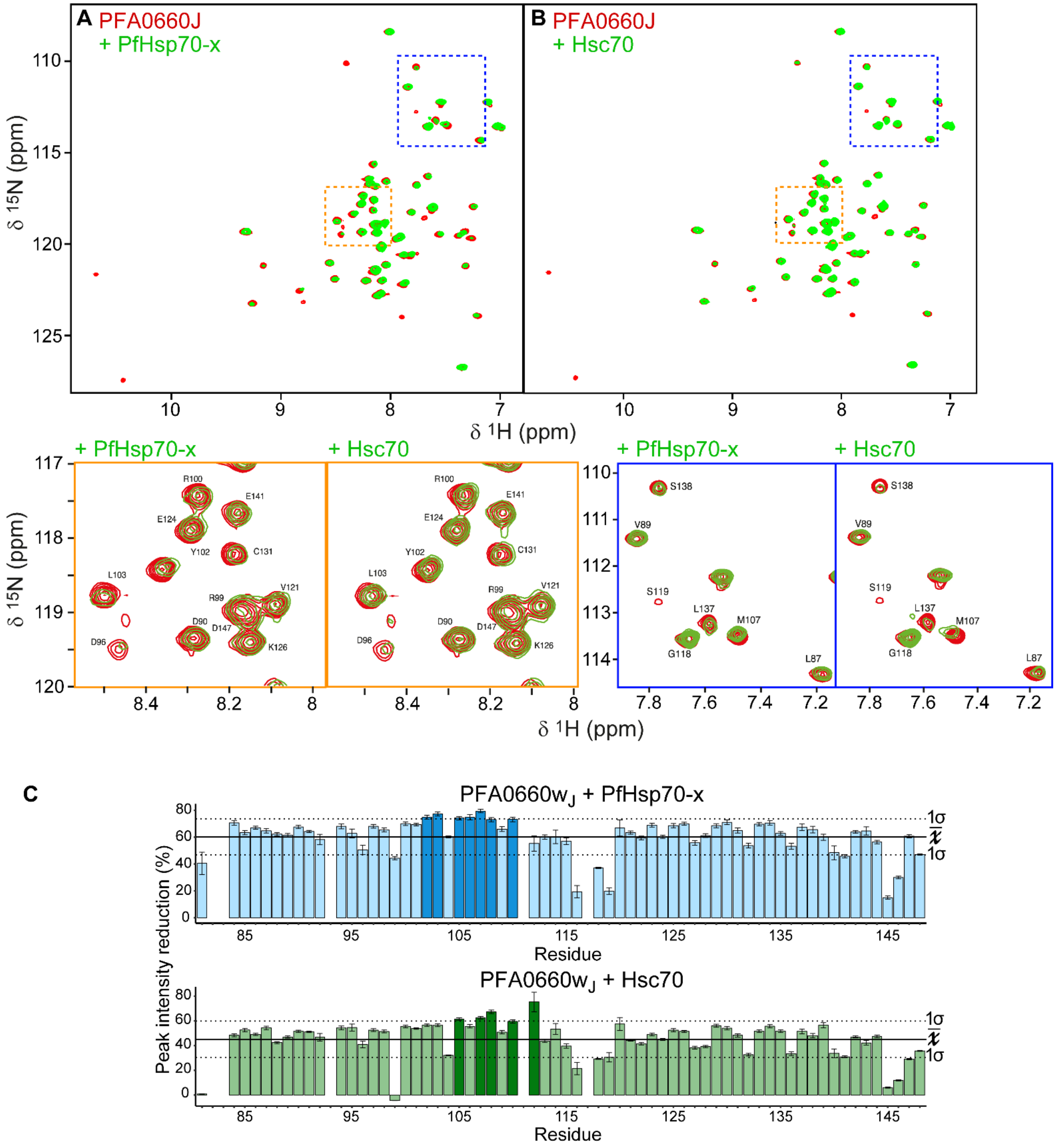

Supplemental Fig. 4: NMR assays of PFA0660wJ interactions with Hsp70 chaperones. (A,B) Overlays of ^{15}N heteronuclear single quantum coherence (HSQC) NMR experiments, showing spectra of 100 μM ^{15}N-labelled PFA0660wJ alone (red), and in the presence of 5-fold molar excess of unlabeled PfHsp70-x (A) or human Hsc70 (B) in green. Top panels show complete protein spectra; bottom panels are magnifications of orange (left) or blue (right) boxed areas. (C) Quantification of fractional reduction in intensity of resonance peaks in PFA0660wJ ^{15}N-HSCQ spectra (from panels A,B) upon titration of PfHsp70-x (top) or human Hsc70 (bottom) as function of co-chaperone amino acid number. The mean intensity reduction and +/- one standard deviation intervals are shown as solid and dashed lines, respectively. Amino acids whose resonance intensities are reduced by more than one standard deviation are shown as darker color bars.
